# MexAB-OprM Efflux Pump Interaction with the Peptidoglycan of *Escherichia coli* and *Pseudomonas aeruginosa*

**DOI:** 10.3390/ijms22105328

**Published:** 2021-05-18

**Authors:** Miao Ma, Margaux Lustig, Michèle Salem, Dominique Mengin-Lecreulx, Gilles Phan, Isabelle Broutin

**Affiliations:** 1 Laboratoire CiTCoM, Université de Paris, CNRS, 75006 Paris, France; mm2303@cam.ac.uk (M.M.); margaux.lustig@parisdescartes.fr (M.L.); michele.salem@u-paris.fr (M.S.); gilles.phan@u-paris.fr (G.P.); 2 Department of Biochemistry, University of Cambridge, Cambridge CB2 1GA, UK; 3 Institute for Integrative Biology of the Cell (I2BC), Université Paris-Saclay, CEA, CNRS, 91198 Gif-sur-Yvette, France; dominique.mengin-lecreulx@i2bc.paris-saclay.fr

**Keywords:** membrane, peptidoglycan, efflux pump assembly, resistance, *Pseudomonas*

## Abstract

One of the major families of membrane proteins found in prokaryote genome corresponds to the transporters. Among them, the resistance-nodulation-cell division (RND) transporters are highly studied, as being responsible for one of the most problematic mechanisms used by bacteria to resist to antibiotics, i.e., the active efflux of drugs. In Gram-negative bacteria, these proteins are inserted in the inner membrane and form a tripartite assembly with an outer membrane factor and a periplasmic linker in order to cross the two membranes to expulse molecules outside of the cell. A lot of information has been collected to understand the functional mechanism of these pumps, especially with AcrAB-TolC from *Escherichia coli*, but one missing piece from all the suggested models is the role of peptidoglycan in the assembly. Here, by pull-down experiments with purified peptidoglycans, we precise the MexAB-OprM interaction with the peptidoglycan from *Escherichia coli* and *Pseudomonas aeruginosa*, highlighting a role of the peptidoglycan in stabilizing the MexA-OprM complex and also differences between the two Gram-negative bacteria peptidoglycans.

## 1. Introduction

The cell wall of Gram-negative bacteria comprises three main layers: the outer membrane lipid bilayer coated by lipopolysaccharides at the exterior; the inner membrane that meets the cytoplasm; and in between the periplasmic matrix where peptidoglycan (PG) confers the mechanical rigidity of the cell wall. Membrane transporters such as efflux pumps span these layers and expulse a wide variety of drugs leading to natural and acquired antimicrobial resistances. Resistance Nodulation-cell Division (RND) family is one of the six families of efflux pumps [1,2], acting as a three-component complex made of an inner-membrane transporter (RND), a periplasmic membrane fusion protein (MFP), and an outer-membrane factor channel (OMF). We focus our interest on the efflux pump MexAB-OprM from *Pseudomonas aeruginosa* *(P. aeruginosa)*, an opportunistic Gram-negative bacterium responsible of severe nosocomial infections and having developed resistance to several families of antibiotics. It has been recently classified as high priority by WHO in antimicrobial research [3] and belongs to the group ESKAPE named by the initials of the pathogens for which there is an urgent need for new treatments. The constitutive RND efflux pumps MexAB-OprM from *P. aeruginosa* and AcrAB-TolC from *Escherichia coli* (*E. coli)* have been extensively studied by different approaches making them archetypal models for the structural and functional comprehension of the efflux pump mechanism. The structure of each protein forming the pump has been solved by X-ray crystallography [4,5,6,7,8,9,10,11,12] and the whole assembly was recently determined by cryo-electron microscopy (cryo-EM) [13,14,15,16]. The comparison of these structures from several homologous pumps, together with in vitro measurements of transport through proteoliposomes [17,18], in cellulo determination of the minimal inhibitory concentration (MIC) of antibiotics for mutant strains [19,20], and the extensive modeling and molecular dynamic calculations [21,22], have led to a more precise understanding of the efflux pathways [23,24,25,26,27,28] and the conformational modifications [14,29,30] necessary to expulse the drugs out of the cell. We know now that the RND protein works as homotrimer with a proton motive force via proton relay amino acids localized in the membranous domain. This proton transfer is combined with the movement of the RND periplasmic domains where each monomer adopts alternatively Loose, Tight, and Close or Open conformations [31], to capture the drug at different entries and push it toward the RND funnel domain [16,32]. The drug then crosses the periplasm by a tunnel formed by the hexameric MFP proteins and is finally expulsed out of the cell through the OMF channel. Even if some hypotheses have been given on the final opening mechanism of the OMF [15,33,34], it is still not clear how and when the MFP and OMF interact. In vitro and in absence of its outer membrane partner, the MFP seems to be unstructured or too flexible to adopt a defined shape around the RND as illustrated by the recent structure of TriABC solved by cryo-EM [35]. In cellulo the tripartite pump must traverse the PG layer to convey the molecules, however, little is known about the relationship between the PG and the pumps. Is the PG a passive mesh made of long glycan chains crosslinked by short peptides or does it make specific interactions with the efflux pump components? If yes, which components are involved in PG recognition? In order to answer these questions, we purified the PG from *E. coli* and *P. aeruginosa* and performed pull-down assays to analyze their interaction with purified OprM and MexA proteins. Here we show that the presence of PG stabilizes the MexA-OprM binary complex formation, highlighting the PG as an important new actor of the assembly of the RND efflux pumps.

## 2. Results

### 2.1. Estimation of the Expected PG Interacting Partners

**Figure 1:**
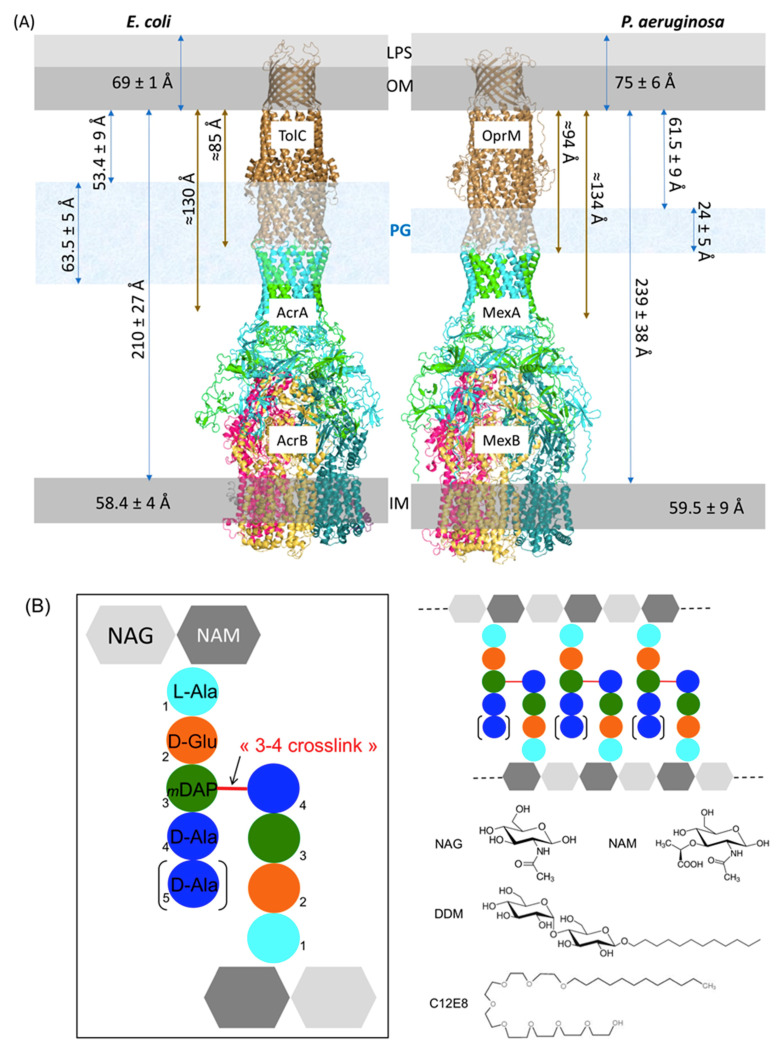
Schematic representation and localization of the PG. (**A**) Structures of the efflux pumps AcrAB-TolC (left) and MexAB-OprM (right) from *E. coli* and *P. aeruginosa*, respectively, presented in their membranous environment. Sizes illustrated by blue arrows come from Cryo-TEM measures performed on high-pressure freezing bacteria sections [36]. Sizes illustrated by brown arrows have been measured on the 3D structures (PDB codes 5ng5 [14] for AcrAB-TolC and 6ta6 [16] for MexAB-OprM). The trimeric OMFs are colored in brown, the MFPs’ trimers of dimers are colored in green and cyan to highlight the different role of the two MFPs forming the dimer, the three monomers forming the RNDs are colored in magenta, yellow and blue depending on their respective functioning states (LTO) [4]. Scale is approximate. Lipopolysaccharides (LPS), outer membrane (OM), peptidoglycan (PG), inner membrane (IM). (**B**) General structure of peptidoglycan organization in Gram-negative bacteria: PG is mainly made of repeating units of disaccharide *N*-acetylglucosamine-β(1-4)-*N*-acetylmuramic acid (NAG-NAM) interconnected by tetra- or pentapeptides cross-linked at amino acid positions 3 and 4 (3-4 crosslinkage). The peptide composition is L-Ala (cyan), D-Ala (blue), D-Glu (orange) and *m*DAP (*meso*-diaminopimelic acid, green). The schematic structures of the two detergents used to solubilize the membrane proteins OprM and MexA are presented for comparison: dodecyl-β-D-maltopyranoside (DDM), which has a similar glucidic component to NAG and NAM, and Octaethylene glycol-monododecyl ether (C_12_E_8_).

We recently solved the structure of the whole MexAB-OprM pump from *P. aeruginosa* by cryo-EM [16] showing a structure of around 230 Å long between the two membranous domains, which is compatible with the size of the periplasm estimated by cryo-transmission electron microscopy (Cryo-TEM) [36]. Matias et al. measured frozen-hydrated sections of *E. coli* and *P. aeruginosa* showing some differences between the two bacteria. The distances between the inner membrane (IM) and the outer membrane (OM) for *E. coli* and *P. aeruginosa* were estimated as 210 Å and 239 Å, respectively, the distances between the PG and the OM were 53 Å and 61 Å, and the thicknesses of the PG were 63 Å and 24 Å. These measurements are not necessarily physiological values as the sample preparation with the used technique resulted in a compression of the bacterial sections that, even if taken into account, increases the uncertainty on the measure. Nevertheless, the large difference of PG width between the two bacteria is remarkable. When adding this width difference to the PG-OM distance, this could reach 116 Å and 85 Å in *E. coli* and *P. aeruginosa*, respectively. If we report these values on the AcrAB-TolC and MexAB-OprM structures (Figure 1), in *P. aeruginosa* the PG would surround the ending part of the periplasmic α-helical coiled-coil domain of OprM and would sweep MexA tips, whereas in *E. coli* the PG would cover both α-helical coiled-coil domains of TolC and AcrA. Anyhow, it appeared clearly that the PG do not interact with the inner membrane RND protein. Consequently, in the present study only OprM and MexA were analyzed for their possible interaction with PG.

### 2.2. Composition Analysis of the PG from E. coli and P. aeruginosa

The peptidoglycan layer is made of linear chains of two alternating amino sugars: *N*-acetylglucosamine (GlcNAc or NAG) and *N*-acetylmuramic acid (MurNAc or NAM), which are attached to a short (3- to 5-residue) amino acids chain. The repeating disaccharide-peptide units are cross-linked via peptide bonds to form a tight mesh barrier (Figure 1B). Even if it is admitted that the PG of *E. coli* and *P. aeruginosa*, both Gram-negative bacteria, present similar composition [37], we decided to quantify the PG composition of the specific *P. aeruginosa* strain used for the binding analysis of this study, PAO1, and to compare it to the PG of *E. coli* that has been extensively studied (Table 1). Besides it is necessary to grow PAO1 in Mueller–Hinton (MH) medium supplemented with salts instead of Luria or Lysogeny Broth (LB) medium to perform in cellulo anti-bioresistance experiments [38]. Therefore, PAO1 strain was grown in both MH and LB media for comparison. For each culture, the PG was purified and submitted or not to enzymatic treatments with α-amylase (E1), to remove high-molecular-weight glycogen, or with both E1 and pronase E (E2), to remove peptidoglycan associated proteins. Each of the six resulting samples were analyzed for their respective composition in the characteristic major components of Gram-negative bacteria, namely NAG, NAM, Ala, Glu, and diaminopimelic acid (DAP) (Figure 1B). The content of Gly is analyzed to verify the absence of protein contaminants although it occurs that a few amount of Gly can be found at position 4 or 5 of the peptide instead of the D-Ala [39]. We show that the treatment with the proteases is necessary to eliminate residual proteins or peptides co-purified with the PG. Nevertheless, in our study the first enzymatic treatment with α-amylase seems to be sufficient for the PG purification. No significant difference was observed between the cultures performed in LB or in MH medium. The relative ratio of NAG/NAM/Ala/Glu/DAP is close to 1/1/1.5/1/1 as expected for *P. aeruginosa*. The small enrichment in Ala is normal as the peptides linking the sugars could vary as tri, tetra and pentapeptides depending on the presence of D-Ala at positions 4 and 5 (Figure 1B) [40,41]. In view of these results, no difference was expected between pull-down experiments performed with PG purified from PAO1 cultures grown in LB and treated with α-amylase only or with the two enzymes E1 and E2. As this hypothesis was verified with our proteins (data not shown), we chose to present only the experiments performed with the PG treated with the two enzymes for clarity.

### 2.3. Pull-Down Analysis of MexA and OprM with Purified PG

#### 2.3.1. Comparison of the PG from *E. coli* and *P. aeruginosa*

**Figure 2:**
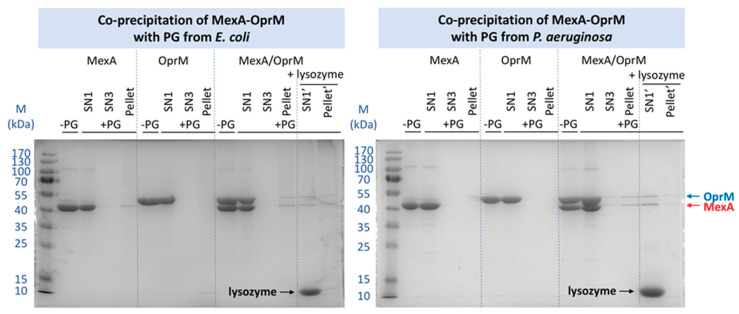
Pull-down experiments performed with the PG extracted from *E. coli* (**left**) and from *P. aeruginosa* (**right**), and with the MexA and OprM proteins in DDM detergent. M: marker of size in kDa; SNn: supernatant of the n washing and centrifugation step; Pellet: final pellet after the 3rd centrifugation; -PG: purified proteins without PG; +PG: pull-down with the PG; + lysozyme: extraction by lysozyme treatment of the remaining proteins from the final pull-down pellet after the three washing steps. SN1’: supernatant after lysozyme treatment after the first centrifugation; Pellet’: pellet after two washing and centrifugation steps.

To analyze the possible interaction between the upper part of the MexAB-OprM efflux pump and the PG, pull-down experiments were performed. The MexA and OprM proteins have been purified from recombinant expression in *E. coli* C43 strain driving our choice to first analyze their interaction with the PG of *E. coli*. The PG has been purified classically [39] from a culture performed on an untransformed C43 strain stopped during the exponential phase of growth. After PG incubation with MexA, OprM, or the complex MexA-OprM at a 2:1 ratio, each sample was extensively washed and the pull-down pellet of PG analyzed by SDS-PAGE, as well as the supernatant of each washing step of the pull-down experiment. As shown in Figure 2, although the PG does not retain most of the proteins, there is still a small proportion of MexA present in the PG pellet, which is not the case for OprM. Concerning the MexA-OprM complex, it clearly appears a supplementary band of OprM, suggesting the complex MexA-OprM-PG affinity to be strong enough to resist to the extensive washing procedure after incubation with the PG, or a stabilization of the MexA-OprM complex in presence of the PG.

The same experiment was performed with the PG purified from PAO1 in exactly the same conditions. No interaction can be observed between OprM and the PG nor between MexA and PG. Nevertheless, when a pre-mixing of the proteins was performed before the pull-down assay, the complex was retained by the PG of *P. aeruginosa* as observed previously with the PG of *E. coli*. For both experiments, the specificity of the pull-down has been verified by releasing MexA and OprM after PG hydrolysis with lysozyme (Figure 2).

#### 2.3.2. Effects of Different Parameters on the Co-Precipitation of MexA-OprM with PG from *P. aeruginosa*

In order to verify the specificity of the stabilized complex, several controls were performed. As MexA and OprM are proteins from *P. aeruginosa*, these experiments were only performed with the PG extracted from this bacterium.

To verify that the presence of PG did not modify the MexA:OprM complex ratio, the experiment was repeated with a 3:1 ratio. The excess of MexA did not modify the relative intensity of the bands in the retained complex (Figure S1), even if more complexes were trapped, indicating that only the 2:1 ratio was stabilized by the PG if referring to our previous intensities calibration [42].

The C-terminus of OprM, corresponding to the 13 last amino acids, was not visible in any structure of OprM resolved by crystallography or cryo-EM. As no functional role of this fragment has been identified so far, it has been hypothesized it could play a role in the PG recognition. The pull-down experiment was repeated with this OprMΔCter protein. No significant difference was detectable in the quantity of MexA-OprMΔCter complex retained by PG compared to the MexA-OprM complex analyzed in the same conditions (see Figure S2).

**Figure 3:**
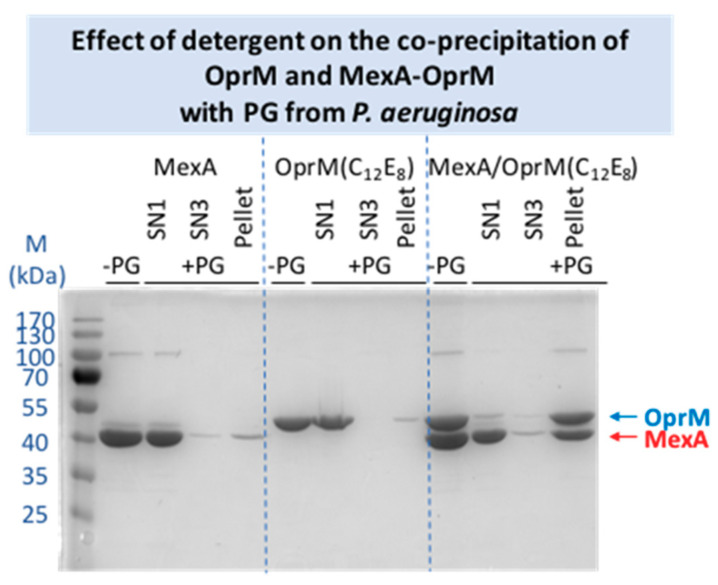
Pull-down experiments performed with the PG extracted from *P. aeruginosa* and with the MexA and OprM proteins after exchange of the DDM detergent for the C_12_E_8_. The same labeling as for Figure 2 have been used.

Another parameter that can modify the result is the nature of the detergent. The chosen detergent, DDM, is the one that has been used to purify and to solve the structure of the isolated proteins and that of the whole pump reconstituted in nanodiscs [11,12,16]. Nevertheless, as the maltoside head group of this detergent can mimic the NAG-NAM disaccharide structure of the PG (Figure 1B), it can be conceived that the presence of the detergent can perturb the interaction of the proteins with the PG. Consequently, a change of detergent was performed for OprM and MexA, replacing DDM by C_12_E_8_, a PEG alkyl ether detergent presenting no similarity with the PG structure (Figure 1B). The co-precipitation with PG was performed in the same conditions as used for the proteins in DDM. As shown on Figure 3, a faint interaction of OprM(C_12_E_8_) and of MexA(C_12_E_8_) with PG is now observed. However, unexpectedly, when the proteins are purified in this detergent the quantity of the retained complex is largely increased, confirming a stabilization of the complex by PG but also revealing an effect of the detergent on this interaction. From the final evaluation of the relative intensity of the bands corresponding to the retained proteins, even though a large quantity of MexA is released after the first washing step (Figure 3), the MexA:OprM pull-down ratio in C_12_E_8_ seems to be comparable to the one observed with DDM detergent. It is not clear if the detergent is directly involved in the interaction with the PG, but the behavior of the protein obviously depends on it. For instance, OprM is less stable in C_12_E_8_ as an important quantity of protein was lost during the detergent exchange. Because the final quantity of retained complex is important, two additional washing steps were added showing further release of MexA compared to OprM. Thus, instability of the membrane proteins in this detergent could be a bias in our interpretation of PG pull-down.

## 3. Discussion

The RND efflux pumps form a tripartite assembly which go through the two membranes of Gram-negative bacteria and the PG. Even if the distances between these three elements are known to fluctuate in spite of direct bonding between OM and PG via lipoproteins, position of PG has been restricted for long at the equatorial region of the OMF protein (see [2] for an example). This is mainly due to the fact the first models of the assembly were built with a direct interaction between the RND transporter and the OMF, suggesting that the PG could not reach the periplasmic coiled-coil helices of the OMF proteins (TolC or OprM) already involved in the binding of the MFP (AcrA or MexA). Since 2015, all the solved cryo-EM structures of AcrAB-TolC [13,14] and MexAB-OprM [15,16] revealed an elongated pump with a tip-to-tip interaction between the OMF and the MFP changing our mind about the position and the possible role of PG in the assembly. Nevertheless, as the PG of *E. coli* is three times thicker than the one of *P. aeruginosa,* it is supposed to cover a large portion of the tripartite assembly, including the coiled-coil domains of TolC and the α-hairpin loops of AcrA together with part of the equatorial domain of TolC, unlike the PG of *P. aeruginosa* that seems to contact the extremities of MexA only (Figure 1A). A recent structure of AcrAB-TolC solved by cryo-tomography in cellulo [43] confirmed the suggested interacting domains of the efflux pump with PG.

Concerning their composition, the two purified PGs are very similar as reported in Table 1. It can be noticed that after the α-amylase action the PG of PAO1 seems to be already free of residual amino-acids contrarily to the PG of *E. coli* that is known to be poorly purified by the sole action of this enzyme. The main difference in crude PG composition is a clear excess of Glu and Ala, especially with PAO1 grown in MH medium. This highlights the importance to get rid of residual interacting proteins as they could alter the binding experiments.

For proteins purified in DDM, when mixed with OprM, no interaction was detected whatever the PG as shown by the absence of the protein in the pellet after extensive washing process, even if a faint band was still detected in the supernatant after the second round of washing, justifying an additional washing step. Concerning MexA, it has been retained with the PG only when using the *E. coli* one. This can be due to the thickness difference between the two PGs. This result indicates a weak interaction that might involve a specific repartition of polar or basic residues on MexA in favor of PG binding. In the in cellulo structure of the *E. coli* efflux pump solved by cryo-tomography, Shi et al. [43] were able to reconstitute images corresponding only to the bottom part of the pump, AcrAB. This bipartite assembly corresponded to 38% of the reconstituted images and presented AcrA in a favorable position to be embedded in the PG. On the contrary they did not mention images corresponding to isolated TolC even if this can also be due to the low resolution of the method. In order to identify the binding sites with PG on the two proteins, they did crosslinking with 3,3′-dithiobis(sulfosuccinimidyl propionate) (DTSSP) followed by mass-spectrometry analysis. They identified several peptides on the two proteins localized in the equatorial and coiled-coil domains of TolC and in the α-hairpin and lipoyl domains of AcrA. When comparing the identified peptides with the equivalent ones in OprM and MexA after sequences alignment, it appears that there is neither conserved residues nor PG binding-motif. The absence of interaction of OprM with the PGs was not intuitive as it is postulated that the OMF is positioned in a waiting state until an RND-MFP complex diffusing in the inner membrane arrives. Nevertheless, it was not excluded that in vitro the detergent dodecyl-β-D-maltopyranoside (DDM) used for proteins purification could prevent the proper interaction of PG by competition, as DDM presents sugar moiety similar to the one in PG unit (see Figure 1B). For comparison, an exchange of detergent to C_12_E_8_ was performed for OprM and MexA, revealing a weak interaction of individual proteins with PG, of the same magnitude as the one observed between MexA (in DDM) and the PG of *E. coli* revealing a significant role of the detergent used to purify the proteins.

Concerning the pull-down experiments performed on the pre-mixed MexA-OprM complex, the two proteins are both retained, suggesting a stabilization of the complex in presence of the PG, whatever the PG origin (*E. coli* or *P. aeruginosa*, Figure 2). Despite the lack of structural information, it was very tempting to consider the last 13 C-terminus residues of OprM to interact with the PG as this part of the protein is rich in polar (Thr, Gln) and charged (Lys) amino acids able to interact with the NAG-NAM or the DAP as observed by Boags et al. [44] in a molecular dynamic study on the interaction of TolR and OmpA with PG. Their calculation resulted in the prediction of a predominant role of the flexible C-terminus of TolR involved in first anchoring of the protein to the PG before making more specific interaction. To explore this hypothesis, the pull-down experiment was repeated with the OprM protein deleted of its 13 last amino acids, not visible in the crystallographic nor the cryo-EM structures, but the use of this truncated OprMΔCter did not modify the result of the pull-down experiment, the quantity of MexA-OprMΔCter complex stabilized by PG being conserved (see Figure S2), thus invalidating a role of OprM C-terminus in the stabilization by PG. Concerning the experiments performed with the MexA-OprM complex after an exchange of detergent, the stabilization of the complex seems largely increased, even if a large amount of MexA is present in the supernatant after the first washing step (Figure 3). This stabilization can be due to the nature and the length of the C_12_E_8_ detergent which is longer than DDM. Nevertheless, a synergistic interaction is also observed when using C_12_E_8_ detergent and the PG seems to rebalance the quantity of protein forming the complex.

This synergistic interaction with PG was previously suggested by Xu et al. [45] when studying the binding of TolC and AcrA with the PG of *E. coli*. Nevertheless, the results were not easy to compare as only the final PG pellet after mixing with TolC and AcrA was analyzed on an SDS-PAGE revealed by Western blot. This stabilization can be interpreted by two different hypotheses. It can be due to specific electrostatic interactions as the helical domains of the two proteins are submitted to large conformation changes during the assembly and opening process that could make particular regions accessible during the process. Nevertheless, it is difficult to know at which step the proteins will be in position to interact with the PG, preventing from a rigorous model building. The second hypothesis could be a geometrical restriction. The structure of a synthetic PG has been solved by NMR [46] and the structure of TolC was docked in the PG holes showing that the protein can enter in the PG without enlargement of the holes by enzymes as the PG pores diameter have been measured to be ≈70 Å. Nevertheless, at that time the PG interacting zone was suggested to be the equatorial domain of TolC measured to be as large as the PG hole. The coiled-coil domain diameter is smaller favoring the insertion of the protein. On the MFP side, the helical tips of the hexamer form a circle larger than the TolC or OprM extremity but still slightly smaller than 70 Å, with an enlargement when in complex with the OMF proteins. So, it can be suggested that MexA do not interact with the PG when uncomplexed as its hexameric tunnel is too small. In presence of OprM, the slightly enlarged tunnel of MexA would be sufficient to contact the PG, stabilizing the complex. It will be difficult to decide between the two hypotheses, but in both cases the PG clearly appears as an important player in the mechanism of the pump assembly.

## 4. Materials and Methods

### 4.1. Purification of the Peptidoglycan

PGs from *E. coli* and *P. aeruginosa* were prepared according to the methods of Mengin-Lecreulx and van Heijenoort [47] with some modifications. Cultures of *E. coli* were performed in LB and cultures of *P. aeruginosa* were performed in LB or in MH media. They were grown at 37 °C under 180 rpm agitation until reaching the middle of the exponential phase at an optical density OD_600nm_ of 0.8. It is important to stop all the cultures at the same growing step, here the exponential phase, as the composition of PG is growth-phase dependent [47,48,49]. A similar protocol was used to purify the PG from *E. coli* or *P. aeruginosa* cultures as follows. Cells are pelleted by centrifugation at 4 °C, 5000× *g* for 30 min then washed with 25 mL of 0.9% NaCl. Cells are pelleted once again by centrifugation at 4 °C, 5000× *g* for 30 min and resuspended in 10 mL of 0.9% NaCl, then added drop by drop in 10 mL of boiling SDS 8% with strong stirring. The mixture is left under boiling conditions for 2 h with stirring then cooled down at room temperature overnight without stirring and finally centrifuged at 200,000× *g*, 20 °C for 30 min to collect the PG sacculus. The pellet is washed at least three times to remove SDS with 6 volumes of warm sterile MilliQ water (30–40 °C), centrifuged at 200,000× *g*, 20 °C for 30 min and the final pellet is resuspended in distilled water or in 10 mM Tris-HCl pH 7.5 if an enzymatic treatment is performed. The pellet might change from white to transparent during the wash. The final PG suspension can be used directly or submitted to digestion with proteases in order to remove the possibly bound proteins. It can be treated with 100 μg/mL α-amylase for 1 h at 37 °C then with 200 μg/mL of preheated pronase E overnight at 37 °C. The digestions are stopped by boiling 30 min in 1 volume of SDS at 4% final concentration. The resulting PG, either collected after the α-amylase digestion or after the second step is then washed as previously described and the final pellet is resuspended in 1 volume of distilled water for further use.

For quantification and composition analysis, a small portion (50 μL) of the sample is hydrolyzed in HCl 6M at 95 °C for 16 h, dried, and solubilized in 500 μL of citrate buffer pH 2.2 before analysis with a Hitachi model 8800 amino acid analyzer (ScienceTec) as described in Barreteau et al. [50]. The characteristic constituents of the PG are *N*-acetylmuramic acid (MurNAc or NAM), diaminopimelic acid (DAP) and *N*-acetylglucosamine (GlcNAc or NAG) for Gram-negative bacteria, with an expected ratio of approximatively 1:1:1 [47].

### 4.2. Cloning and Purification of the Analyzed Proteins

Wild type OprM and MexA were cloned and purified following the protocol described in [11,42] with minor modifications. OprM deleted of the flexible C-terminus residues 473–485 (OprM∆Cter) was generated as 5′-NdeI and 3′-XmaI fragment using polymerase chain reaction (PCR) with PAO1 genome as template. A 6-His tag was included at the C-terminus for Ni-NTA affinity purification. Forward and reverse primers are GGAATTCCATATGAAACGGTCCTTCCTTTCC and TCCCCCCGGGTCATGATGATGATGATGGTCAGGTCTGCTGGTTCCAGCCGCCGCCGA, respectively. The PCR fragment was then inserted into pBAD33 expression vector as described in [11]. Heterologous expression of OprM full-length or OprMΔCter (deletion of the last 13 C-terminus residues 473–485) inserted in a pBAD33 plasmid is performed in a *E. coli* C43 strain deleted of *acrB* gene. The preculture is performed at 37 °C in LB medium under 200 rpm agitation and inoculated at OD_600nm_ = 0.05 in LB medium containing 25 μg/mL of chloramphenicol. Cell were grown to OD_600nm_ = 0.1 at 37 °C, 200 rpm, then cooled down to 20 °C. Cells were induced at OD_600nm_ = 0.7 with arabinose (0.02% final, *w*/*v*) and grown overnight at 20 °C before centrifugation. The cell pellet was resuspended in TBS buffer (20 mM Tris-HCl pH 8, NaCl 150 mM) and broken by the use of a cell disrupter (CellD) at 30,000 psi (2400 Bar) before centrifugation at 10,000× *g* for 20 min. The supernatant was diluted down to 1 mg/mL and solubilized into TBS with DDM 2%, imidazole 10 mM during 1 h at room temperature. The insoluble fraction is pelleted by ultracentrifugation at 100,000× *g* for 1 h at 4 °C and the solubilized fraction is loaded on a Ni-NTA column pre-equilibrated in TBS with 0.05% DDM (*w*/*v*). The column is washed with the same buffer supplemented with 20 mM imidazole pH 8. The protein is eluted between 100 to 250 mM imidazole, concentrated and injected on a Superdex 200 gel-filtration column equilibrated with the same buffer without imidazole. OprM is eluted as a trimer and concentrated at 5 mg/mL before use. MexA is purified following the protocol described in [17] which is similar to the one used for OprM at the exception of the culture, which is performed in TB medium, and grown at 30 °C during 2.5 h after induction.

The exchange of DDM detergent for C_12_E_8_ has been performed on Ni-NTA by reloading the pure proteins on the affinity column. After extensive washing with TBS with 0.025% C_12_E_8_ (*w*/*v*) and 20 mM imidazole pH 8, the samples were eluted in the same buffer at 500 mM imidazole. The samples were not further purified but several cycles of concentration and dilution on amicon® 10K were performed to get rid of the imidazole. Samples are finally resuspended in TBS with 0.025% C_12_E_8_ (*w*/*v*) and 300 mM NaCl before use for the pull-down experiments.

The purify of the proteins can be evaluated by analyzing the well without PG (-PG) on Figure 2, Figure 3, and Figure S2.

### 4.3. Pull Down with PG

The purified PG is insoluble in water. It is vortexed before mixing with the proteins. The pull-down experiment is performed with 50 nmoles of PG (in terms of DAP content, see paragraph 4.1) in 100 μL of the following buffer: 20 mM Tris-HCl, pH 8.0, 300 mM NaCl, 0.05% DDM. The PG:protein ratio is 100:1 for OprM, 100:2 for MexA, 100:2:1 for MexA-OprM to account for the 2:1 MexA:OprM ratio in the efflux pump assembly. The mix is incubated on a rolling wheel for 1 h at room temperature before centrifugation at 18,000× *g* for 5 min. The supernatant is kept for further analysis on an SDS-PAGE. The pellet is washed with 2 volumes of buffer before centrifugation. This washing–centrifugation cycle is repeated 3 times. The final pellet is resuspended in 1 volume of buffer. The pure protein, the three supernatants and the final pellet are analyzed on a 12% SDS-PAGE after 5 min boiling. The experiment was repeated 5 times. For 2 experiments the final pellet was treated with lysozyme to release the pulled-down proteins. The pellet is resuspended in 1 volume of buffer with 1 mg/mL final concentration of lysozyme and incubated on a rolling wheel for 1.5 h at room temperature before centrifugation at 18,000× *g* for 5 min. The pellet is washed with 2 volumes of buffer and solubilized in 1 volume. The first supernatant and the final solubilized pellet after the second washing step are analyzed on a 12% SDS-PAGE.

## 5. Conclusions

The MexAB-OprM efflux pump spans the complete cell wall of *P. aeruginosa*, crossing the peptidoglycan in the periplasmic compartment. Here we analyzed the possible specific interaction of the MexA and OprM proteins with the PG purified from *E. coli* or *P. aeruginosa* revealing, when proteins are purified in DDM, no interaction for OprM and an interaction of MexA only with the PG of *E. coli* that is three times thicker in vivo than the one of *P. aeruginosa*. Nevertheless, previous association of the two proteins before analysis reveals a stabilization of the complex–PG interaction in both cases. This is further reinforced with the PG of *P. aeruginosa* after the exchange of DDM detergent for C_12_E_8_ for the two proteins. Even if no covalent binding is expected, this synergistic interaction could correspond to a required step of the assembly mechanism, suggesting the PG to be an important actor of the process. It could help to stabilize MexA to keep it in the proper quaternary structure in order to increase the speed of the pump formation with OprM present in the opposite membrane.

## Figures and Tables

**Table 1:** PG composition of *E. coli* and *P. aeruginosa* bacterial strains normalized to the NAG concentration.

	NAG	NAM	Ala	Glu	DAP	Gly
*E. coli*						
crude PG extract	1	0.93	6.35	5.81	0.98	3.19
purified PG	1	0.98	1.82	1.13	1.03	0.19
*P. aeruginosa*						
LB medium crude PG extract	1	1.02	2.38	1.78	1.11	0.31
LB medium PG treated by E1	1	0.98	1.57	1.06	0.99	0.04
LB medium PG treated by E1+E2	1	0.98	1.60	1.16	1.00	0.08
MH medium crude PG extract	1	0.94	2.70	2.21	1.11	0.63
MH medium PG treated by E1	1	1.03	1.58	1.09	1.02	0.03
MH medium PG treated by E1+E2	1	0.98	1.60	1.25	1.05	0.19

## Supplementary Materials

Supplementary Materials can be found at https://www.mdpi.com/article/10.3390/ijms22105328/s1. Figure S1: Pull-down experiments performed with the PG extracted from *P. aeruginosa* and with the MexA and OprM proteins mixed at two different ratios, 2:1 and 3:1., Figure S2: Pull-down experiments performed with the PG extracted from *P. aeruginosa* and with the MexA and OprM modified proteins, OprM∆Cter corresponding to a truncation of 13 residues at its C-terminus.

## Abbreviations

PG	peptidoglycan
NAM	*N*-acetylmuramic acid
NAG	*N*-acetylglucosamine
DAP	diaminopimelic acid
