# Supplementary material for: MexAB-OprM Efflux Pump Interaction with the Peptidoglycan of Escherichia coli and Pseudomonas aeruginosa
Source: Int J Mol Sci. 2021 May 18;22(10):5328. doi: 10.3390/ijms22105328 (PMC8158685; doi:10.3390/ijms22105328)
Supplement: Supplementary file 1 [file ijms-22-05328-s001.zip › ijms-1203436-supplement.pdf]

Supplementary Material

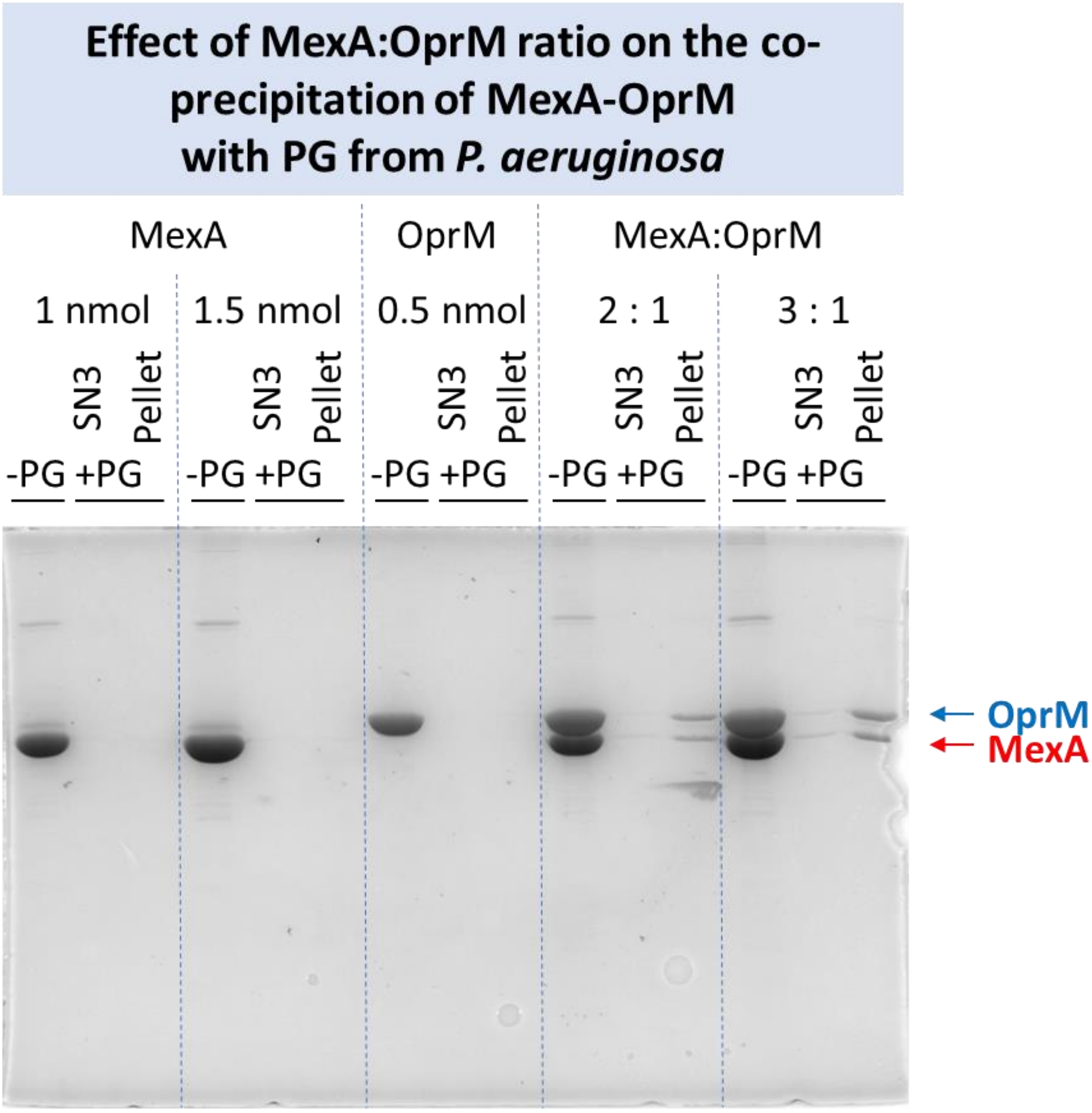

Figure S1. Pull-down experiments performed with the PG extracted from *P. aeruginosa* and with the MexA and OprM proteins mixed at two different ratio, 2:1 and 3:1. No marker was loaded in order to be able to analyze the two ratio on the same SDS gel. SN3 and Pellet: supernatant and Pellet of the 3rd washing and centrifugation step; -PG: purified proteins without PG; +PG: pulldown with the PG.

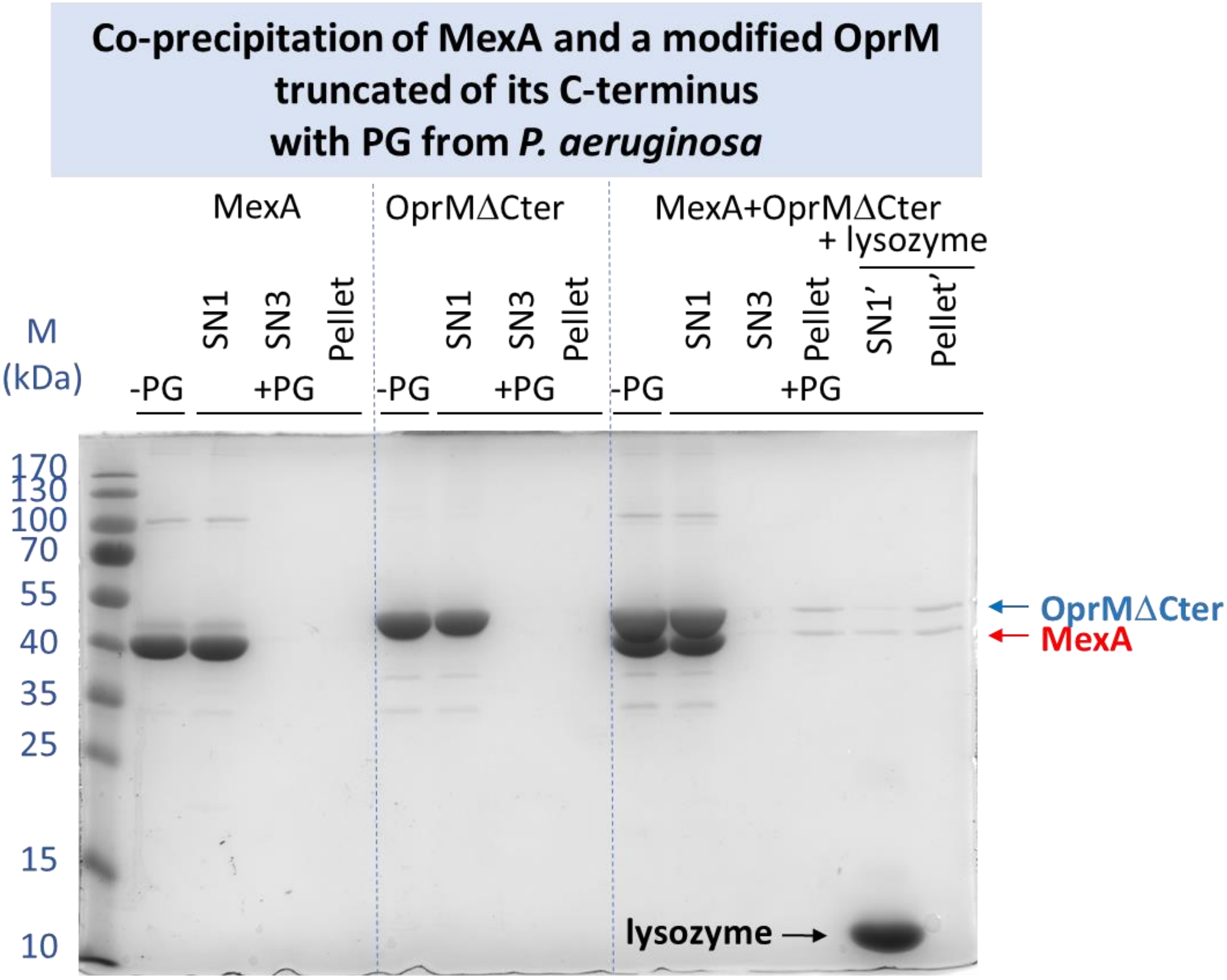

Figure S2. Pull-down experiments performed with the PG extracted from *P. aeruginosa* and with the MexA and OprM modified proteins, OprMDCter corresponding to a truncation of 13 residues at its C-terminus. M: marker of size in kDa; SNn: supernatant of the n washing and centrifugation step; Pellet: final pellet after the 3rd centrifugation; -PG: purified proteins without PG; +PG: pull-down with the PG; + lysozyme: extraction by lysozyme treatment of the remaining proteins from the final pull-down pellet after the three washing steps. SN1': supernatant after lysozyme treatment after the first centrifugation; Pellet': pellet after two washing and centrifugation steps.
